# Midterm results of tricuspid annuloplasty with a homemade PTFE flexible ring

**DOI:** 10.1186/1749-8090-10-S1-A189

**Published:** 2015-12-16

**Authors:** Salim Chibane, Abdelmalek Bouzid, Mohamed Atbi, Halima Larbi, Ramdane Amar Ould Abderahmane

**Affiliations:** 1 Department of Cardiac Surgery, 1st November Hospital, Oran 31000, Algéria

## Background/Introduction

Tricuspid regurgitation is a serious valvulopathy with a significant impact on long term survival. Several techniques have been described to address it, including suture techniques and ring techniques. All have the same purposes of favoring leaflet cooptation, reducing the size of the tricuspid annulus and stabilize the reparation to preclude further re expansion and dilatation of the annulus.

## Aims/Objectives

The purpose of this study is to evaluate the outcome of tricuspid annuloplasty with a PTFE linear strip.

## Method

From February 2009 to May 2013, seventy-two patients underwent tricuspid annuloplasty for functional tricuspid regurgitation (TR). In 32 patients, tricuspid annuloplasty was done with a linear strip of PTFE. All patients had a left valvulopathy associated. The mean age was 42.7 years and 15.6% were reoperations. Right heart failure was present in 19% of patients on admission and 56% had atrial fibrillation. Ten patients (31.2%) were symptomatic (NYHA class 3 and 4) and 22 patients (69%) had significant tricuspid regurgitation (grade 3-4).

## Results

The mean follow-up was 18 months (min 4 months, max 53 months). Early and late mortality were 9.1% and 6.8% respectively. At last follow-up, all patients had few or no symptoms (NYHA class 1or 2). Eighteen patients (66.7%) had TR grade 0-1, five patients (18.5%) had TR grade 2 and four patients (14.8%) had TR grade 3 but remained asymptomatic under medical treatment. No patient was reoperated. On echocardiography, there was a significant reduction in the size of the right ventricle and tricuspid annulus with a mean tricuspid gradient of 3 mm Hg at the last follow-up.

## Discussion/Conclusion

Tricuspid annuloplasty with a PTFE linear strip is effective for the treatment of functional tricuspid regurgitation with good midterm results. This technique is a good alternative of annuloplasty in case of unavailability of a prosthetic ring especially in developing countries. A long-term follow up is needed to see if these results are sustainable.

